# Think for Yourself

**Published:** 1871-05

**Authors:** 


### THINK FOR YOURSELF.

A GREAT many preachers and old women have taken to writing for newspapers and magazines, at a dollar an acre, about health; and peripatetic doctors, who can't earn their salt at home, are strolling through the country lecturing the people, at two cents a ticket, as to the best means of living indefinitely long without ache or pain or symptom. A clerical friend of ours, after writing for all sorts of papers on all sorts of subjects connected with drugs, druggers, physiology, syntax, and prosody, and after seeing a little more of the world, and of himself, and of whole facts, wrote lately of the past, "I was an ass then." Sooner or later these teachers of the public may arrive at the same conclusion as to themselves. Meanwhile if people will fool away their money in listening to wandering lecturers and amateur writers on health and disease, they would do well to think for themselves, and weigh every assertion by their own personal experience.

If an ignoramus gets up and announces that milk is nature's food, good for the old and young, wholesome for all mankind, you may, if you have had no personal experience to the contrary, and have no means of comparing notes with older persons, make the experiment and drink a pint of fresh, rich milk at each meal for ten days, and if you do not lose your appetite, have a bad taste in the mouth of mornings, with less free bodily functions and more or less nausea, then you may conclude that you at least are proof against the

UNWHOLESOMENESS OF MILK

as a large item in our daily food. But multitudes of people have found out from their own observation, that although they had a passion for fresh, pure, rich farm-house milk, it did not agree with them ; that it made them bilious, gave a coated tongue in the morning, caused a sickish feeling during the day, and tended to promote constipation. Laboring men who spend most of their time in the open air, may use sweet milk largely, and nevertheless remain well and strong; but such a thing is impossible for persons who spend a large portion of their time in-doors, who are sedentary. Not long since a person lectured in New York and elsewhere against the use of

TEA AND COFFEE.

His knock-down argument was, that when in Paris the use of them so much intoxicated him that he had to retire for the evening. I could say in reply, that when I was in Paris, a hundred years ago, more or less, I used tea and coffee, and didn't have to retire at all; and no doubt the experience of ninety-nine readers out of a hundred would be the same as mine. But the very idea of a man setting himself up as a teacher of health, and making his feelings and habits and experiences the rule and guide and law for all, is utterly absurd.

The same lecturer stated that men could not live without water as a beverage ; when the truth is, there are millions of people, especially in wine countries, who do not take one swallow of cold water in a week. A man in health may seldom think of drinking a swallow of water from daylight till dark of any day, and very little of any other liquid. But in proportion as persons take exercise in the open air, or engage in any form of work which induces perspiration, water or some other liquid is desirable, in order to keep the blood sufficiently diluted to flow healthfully. A large portion of the food we eat is water, especially vegetables and fruits and fishes, hence cold water, as a drink of itself, is not a daily necessity; a man may have good health, and not take a swallow of cold water in a month.

THE BEST DRINK

for sedentary persons is hot tea and coffee of moderate strength, and that at meal times; it is the best drink at meals for all who are past fifty years of age; all such will live longer by the use of these as a beverage than by the use of cold water as a beverage. The fact is observable with the naked eye, that a glass of cold water during a meal arrests digestion instantly; which process can only be resumed when this cold water, of fifty degrees, has been long enough in the stomach to absorb heat sufficient from the body to warm it up to a hundred degrees. That a hot cup of tea at a meal does revive a person who is old, or feeble, or cold, or tired, is the daily experience of millions. If a healthy person greatly desires a drink of cold water during a meal, let him take it by all means; it is only intended to inculcate the general idea for the masses, that the habitual and large use of cold water at a meal, or within half an hour before or after, is not wise, is not safe, and that a cup of hot drink is always safe, is always beneficial, is always reviving; if now and then there is an exception, it should be set down as a peculiarity or an idiosyncrasy.

